# Bimatoprost Induced Serous Macular Detachment after Cataract Surgery

**DOI:** 10.1155/2016/7260603

**Published:** 2016-11-13

**Authors:** Swapnil Parchand, Subashini Kaliaperumal, Amit Kumar Deb, K. Ramesh Babu, Renuka Srinivasan

**Affiliations:** Department of Ophthalmology, Jawaharlal Institute of Postgraduate Medical Education and Research (JIPMER), Pondicherry 605006, India

## Abstract

We report a case of bimatoprost induced serous macular detachment and choroidal folds following uneventful cataract surgery. A 66-year-old male using topical bimatoprost in both eyes for open angle glaucoma underwent uneventful cataract surgery in the right eye. Postoperatively, he was restarted on topical bimatoprost and antibiotic-steroids combination drops. One week after surgery, he presented with conjunctival hyperemia, serous macular detachment, and choroidal folds at the posterior pole. Fundus fluorescein angiography showed perifoveal leaks in early stage with pooling of dye in late stage. Discontinuation of bimatoprost led to resolution of serous detachment and choroidal folds within 3 weeks with significant improvement in visual acuity. Occurrence of serous macular detachment and choroidal folds in this case could be probably related to the proinflammatory property of bimatoprost. Hence, it should be used with caution in the immediate postoperative period after cataract surgery.

## 1. Introduction

Bimatoprost is a synthetic prostamide with ocular hypotensive effect acting on the uveoscleral pathway [1]. It triggers a cascade of tissue remodeling enzymes, such as metalloproteinases and transcription factors such as c-Fos, thereby degrading collagen. This opens the intercellular spaces for fluid drainage and ultimately leads to an increase in uveoscleral flow rates. In most countries, prostaglandin analogs are prescribed as first-line and first-choice treatment for glaucoma patients with good compliance due to single dose daily regimen. Though it has good IOP lowering capacity, it is associated with few side effects like conjunctival hyperemia, increased pigmentation in the periocular skin and iris, hypertrichosis, increased severity and recurrence of herpetic keratitis, and cystoid macular edema [2]. After a thorough literature search, we came across only one report of development of serous macular detachment (SMD) induced by bimatoprost in a patient with Sturge-Weber syndrome with choroidal hemangioma [3]. We here report bimatoprost induced serous macular detachment following uneventful cataract surgery in an adult with primary open angle glaucoma.

## 2. Case Report

A 66-year-old man with primary open angle glaucoma was planned for right eye (RE) cataract surgery for nuclear sclerosis grade 3. Best-corrected visual acuity was 6/24 in the RE and 6/12 in the left eye (LE). His intraocular pressure (IOP) was controlled by topical bimatoprost (0.3 mg/mL) in both eyes (BE) for 4 years. The cup-disc (C : D) ratio was 0.7 in BE. He did not have any systemic illness. He underwent uneventful phacoemulsification with implantation of posterior chamber intraocular lens (PCIOL) in the RE. On the first postoperative day, BCVA in RE was 6/12 and IOP was 21 mm Hg. Anterior segment examination revealed well-apposed incision, clear cornea, mild anterior chamber inflammation with few cells, and well-centered PCIOL in the bag. Fundus examination revealed C : D ratio of 0.7 and normal macula. He was prescribed topical gatifloxacin 0.3%-prednisolone 1% combination 6 times a day and restarted on topical bimatoprost at bedtime.

**Figure 1:**
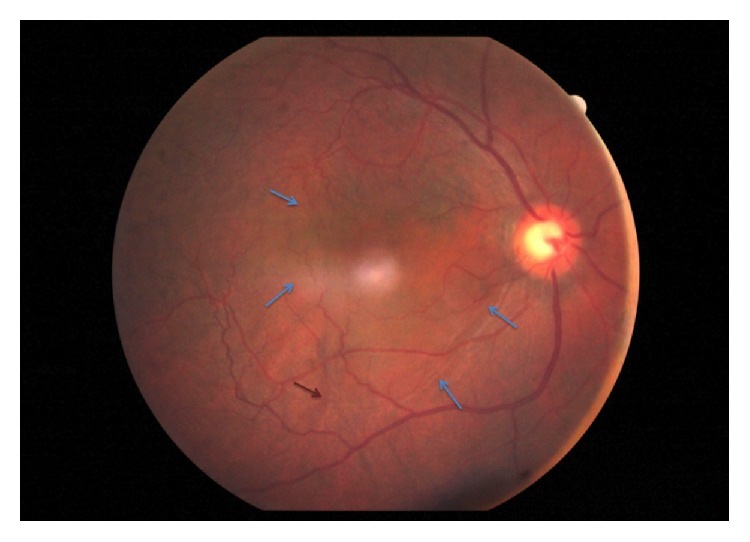
Fundus picture of the right eye showing large serous macular detachment (SMD) (blue arrow) and choroidal folds (brown arrow) at the posterior pole.

**Figure 2:**
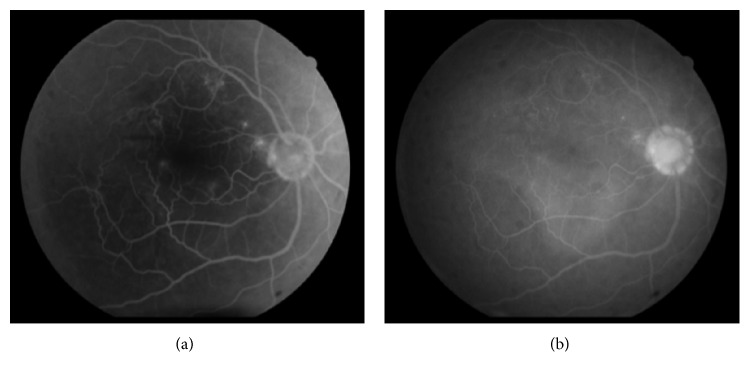
Fundus fluorescein angiography of the same eye in venous phase showing multiple focal leaks at the macula (a) and pooling of dye in the subretinal space in late phase (b).

**Figure 3:**
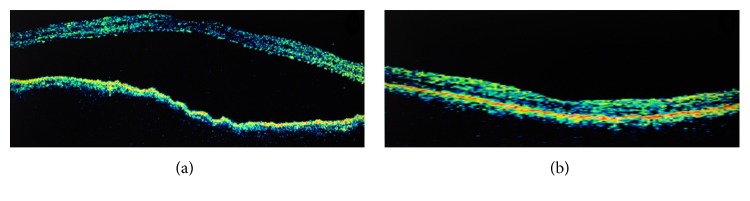
(a) OCT raster line scan of the right eye showing the presence of large SMD with underlying choroidal folds. (b) OCT raster line scan of the same eye showing resolution of SMD and choroidal folds.

He returned on the seventh postoperative day with sudden onset decreased vision in the eye operated on for 2 days. Examination revealed BCVA of counting finger at 1 meter in RE and IOP 12 mm Hg on topical bimatoprost. Anterior segment revealed clear cornea, mild anterior chamber inflammation, and well-centered PCIOL. Fundoscopy showed large serous macular detachment (SMD) and choroidal folds at the posterior pole (Figure 1). Optical coherence tomography (OCT) examination confirmed the presence of large SMD with central macular thickness (CMT) of 830 microns and underlying choroidal folds in the RE (Figure 3(a)). Fundus fluorescein angiography showed multiple focal leaks at the macula in early phase with pooling of dye in late phase in the area corresponding to SMD (Figure 2). We considered SMD and choroidal folds probably as a side effect of bimatoprost and hence it was stopped and replaced by topical timolol 0.5% twice a day and topical antibiotics-steroid combination was continued at 4 times a day. After 1 week of stopping bimatoprost, BCVA improved to 6/60 with a decrease in the size of SMD on OCT. Complete resolution of serous macular detachment and choroidal folds was noted clinically and on OCT (CMT: 210 microns) (Figure 3(b)) at 3 weeks with BCVA improving to 6/9. IOP was well controlled on topical timolol BD dose. At the last follow-up at 2 years, the patient was maintaining BCVA of 6/9 and IOP of 12 mm Hg on topical timolol drops without any recurrence of SMD.

## 3. Discussion

The role of prostaglandins in inflammation has been widely documented. The intraocular inflammatory effects, namely, aqueous cell, flare, and miosis, are seen with the administration of large doses of prostaglandins but have not been reported with the doses used for ocular hypotensive response [2]. Absorptive transport systems of the ciliary processes remove most of the topically applied prostaglandins and other eicosanoids from the anterior chamber. This prevents the drug from causing retinal and choroidal complications. In an animal experiment, it was observed that, in early postoperative period, prostaglandins F2 alpha analogs can trigger the release of endogenous prostaglandins in the eye [4]. It has been suggested that prostaglandins can mimic vascular endothelial growth factor and vascular permeability factor, inducing vascular permeability, and thus can lead to breakdown of both blood-aqueous and blood-retinal barrier [5]. Thus, in a situation like recent cataract surgery which is associated with breakdown of blood-aqueous barrier due to the release of various inflammatory mediators including prostaglandins, even low concentration doses required for ocular hypotensive response can further affect the vascular permeability. Bimatoprost is known to cause conjunctival hyperemia by inducing endothelial derived nitric oxide-mediated vasodilation. Kiel suggested that stimulation of endothelial nitric acid might decrease vascular resistance and increase choroidal blood flow causing choroidal congestion [6]. We hypothesize that disturbance in the choroidal circulation due to bimatoprost led to the breakdown of retinal pigment epithelial (RPE) barrier with subsequent leakage of fluid from the choroid into the subretinal space. Despite being on bimatoprost for four years before cataract surgery, our patient did not show any evidence of intraocular side effects of the drug but when he was restarted on bimatoprost in an inflamed eye after cataract surgery he developed SMD and choroidal folds and subsequently cessation of bimatoprost resulted in the resolution of SMD in our patient within 3 weeks. Also, in our case, there was no other cause that could have led to SMD. This event is classified for the score of 5 on Adverse Drug Reaction (ADR) probability scale suggesting that bimatoprost is probably responsible for the SMD in the early postoperative period in our case [7].

This case demonstrates that topical bimatoprost might be responsible for the development of serous macular detachment in an inflamed eye. Hence, as cataract surgeons, we need to be aware of this complication and try to avoid prostaglandin usage at least in the immediate postoperative period.

## References

[1] Toris C. B., Gabelt B. T., Kaufman P. L. (2008). Update on the mechanism of action of topical prostaglandins for intraocular pressure reduction. *Survey of Ophthalmology*.

[2] Alm A., Grierson I., Shields M. B. (2008). Side effects associated with prostaglandin analog therapy. *Survey of Ophthalmology*.

[3] Addison P. K. F., Papadopoulos M., Nischal K. K., Hykin P. G. (2011). Serous retinal detachment induced by topical bimatoprost in a patient with Sturge-Weber syndrome. *Eye*.

[4] Yousufzai S. Y. K., Ye Z., Abdel-Latif A. A. (1996). Prostaglandin F(2*α*) and its analogs induce release of endogenous prostaglandins in iris and ciliary muscles isolated from cat and other mammalian species. *Experimental Eye Research*.

[5] Vinores S. A., Sen H., Campochiaro P. A. (1992). An adenosine agonist and prostaglandin E1 cause breakdown of the blood-retinal barrier by opening tight junctions between vascular endothelial cells. *Investigative Ophthalmology & Visual Science*.

[6] Kiel J. W. (2000). Endothelin modulation of choroidal blood flow in the rabbit. *Experimental Eye Research*.

[7] Naranjo C. A., Busto U., Sellers E. M. (1981). A method for estimating the probability of adverse drug reactions. *Clinical Pharmacology and Therapeutics*.

